# Supplementary material for: i-Motif folding intermediates with zero-nucleotide loops are trapped by 2′-fluoroarabinocytidine via F···H and O···H hydrogen bonds
Source: Commun Chem. 2023 Feb 16;6:31. doi: 10.1038/s42004-023-00831-7 (PMC9935537; doi:10.1038/s42004-023-00831-7)
Supplement: Supplementary file 2 — Supplemental Information [file 42004_2023_831_MOESM2_ESM.pdf]

Supplementary Information

i-Motif folding intermediates with zero-nucleotide loops are trapped by 2′-fluoroarabinocytidine *via* F···H and O···H hydrogen bonds

Roberto El-Khoury, Veronica Macaluso, Christopher Hennecker, Anthony K. Mittermaier, Modesto Orozco, Carlos González, Miguel Garavís*, Masad J. Damha*

Outline:

A. Supplementary Figures **p2**

Supp. Figure 1. Native gel electrophoresis of RA/SA ON0 and ON5. p2
Supp. Figure 2. CD spectra of SA and RA ON0. p2
Supp. Figure 3. ^{1}H NMR spectra of RA ON5 at different pH. p3
Supp. Figure 4. Native gel electrophoresis of RA/SA ON4a-ON4e. p4
Supp. Figure 5. CD spectra of RA ON5 and ON4a-ON4e. p4
Supp. Figure 6. ^{1}H NMR spectra of RA/SA ON5 and ON4a-ON4e. p5
Supp. Figure 7. NOESY, TOCSY and ^{19}F-^{1}H-HOESY of RA ON4b. p6
Supp. Figure 8. DQF-COSY of RA ON5 and Karplus curves used for analysis. p7
Supp. Figure 9. ^{19}F-coupled and -decoupled 1D ^{1}H-NMR of SA ON4b and HC3. p8
Supp. Figure 10. NCI plots for residues **C6** and **C2′**. p9
Supp. Figure 11. NCI plots for residues **C2** and **C6**′. p10
Supp. Figure 12. NCI plots for residues **C3** and **C5**′. p11
Supp. Figure 13. NCI plots for residues **C5** and **C3**′. p12
Supp. Figure 14. Four-cytosine model system for QM calculations. p12
Supp. Figure 15. NCI plots and reduced density gradient surface for **C6** and **C2′**. p13
Supp. Figure 16. NCI plots and reduced density gradient surface for **C2** and **C6′**. p14
Supp. Figure 17. NCI plots and reduced density gradient surface for **C3** and **C5′**. p15
Supp. Figure 18. NCI plots and reduced density gradient surface for **C5** and **C3′**. p16
Supp. Figure 19. Kinetic simulations of ON0 and ON5. p17
Supp. Figure 20. ^{19}F-coupled and -decoupled 1D ^{1}H-NMR of RA ON5. p17

B. Supplementary Tables **p18**

Supp. Table 1. Chemical shifts of the proton and fluorine signals of RA ON5. p18
Supp. Table 2. Experimental constraints and calculation statistics of RA ON5. p18
Supp. Table 3. Average pseudorotation parameters of RA ON5 i-motif structure. p19
Supp. Table 4. Average dihedral angles and order parameters of RA ON5 struct. p10
Supp. Table 5. Kinetic and thermodynamic parameters from TH traces. p20

C. Supplementary Methods **p21**

D. Supplementary References **p23**

A. Supplementary Figures

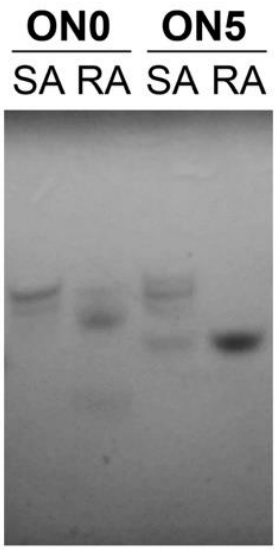

Supplementary Figure 1. Non-denaturing gel electrophoresis of rapid, snap-cooled (RA) and slow-annealed (SA) ON0 and ON5.

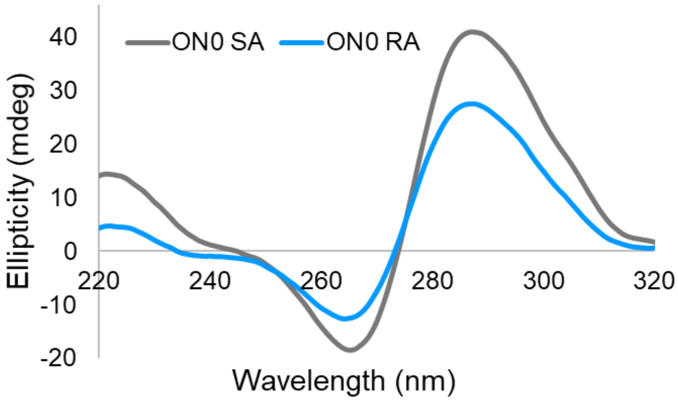

Supplementary Figure 2. CD spectra of rapid and slow annealed ON0 (100μM, 5°C).

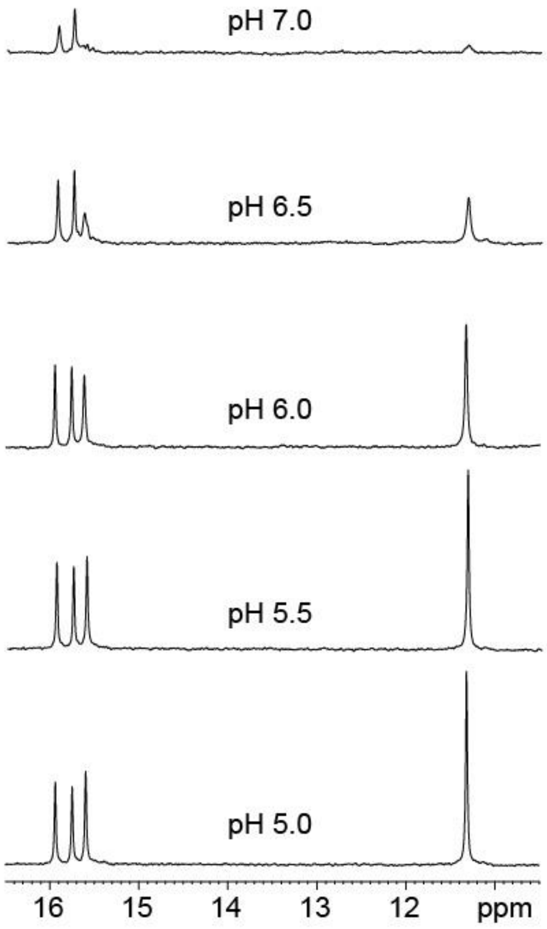

Supplementary Figure 3. Imino region of the ^{1}H-NMR spectra of ON5 at different pH and 5 ºC.

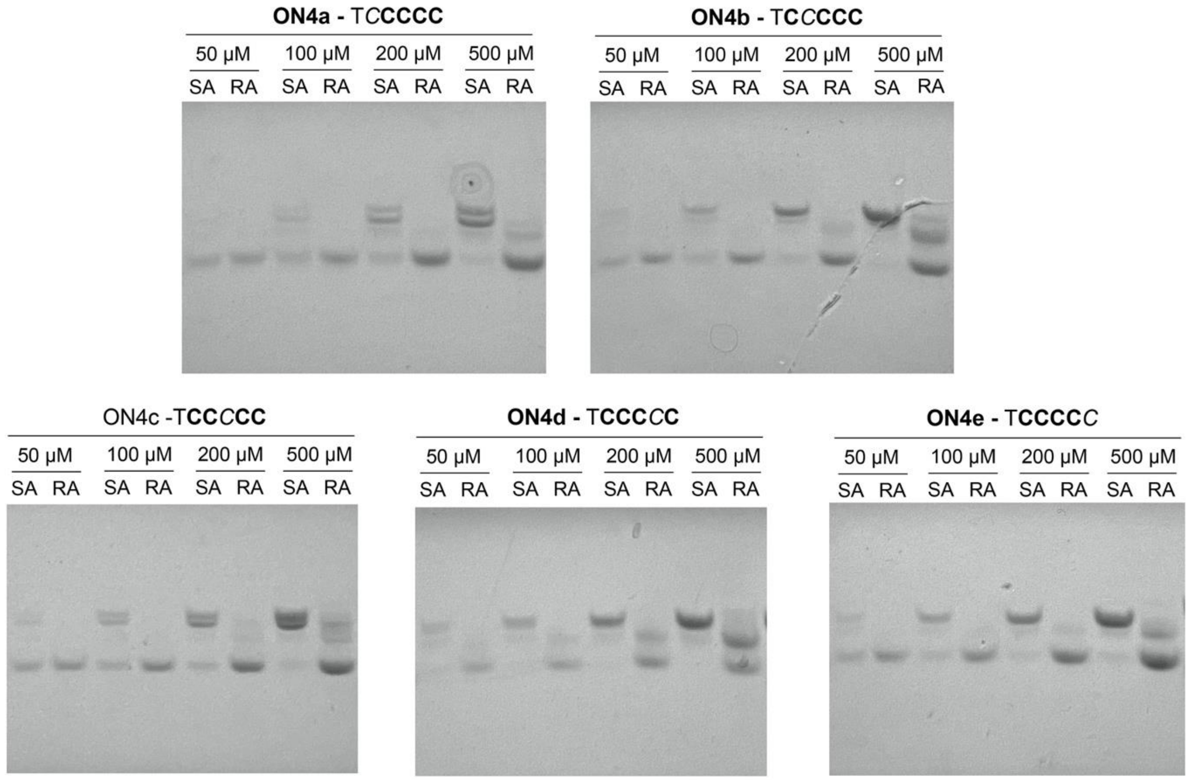

Supplementary Figure 4. Non-denaturing gel electrophoresis of rapid, snap-cooled (RA) and slow-annealed (SA) ON4a-ON4e across concentrations in the range of 50-500 µM.

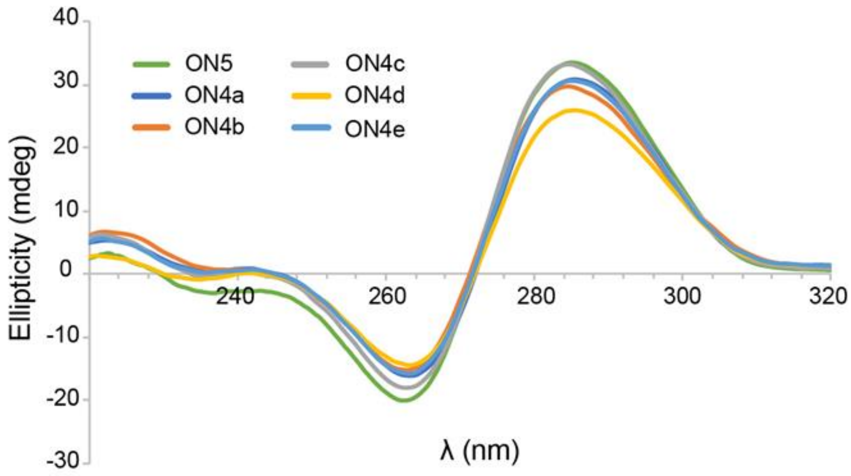

Supplementary Figure 5. CD spectra of rapid, snap-cooled (RA) ON5 and ON4a-ON4e (100 µM, 5°C).

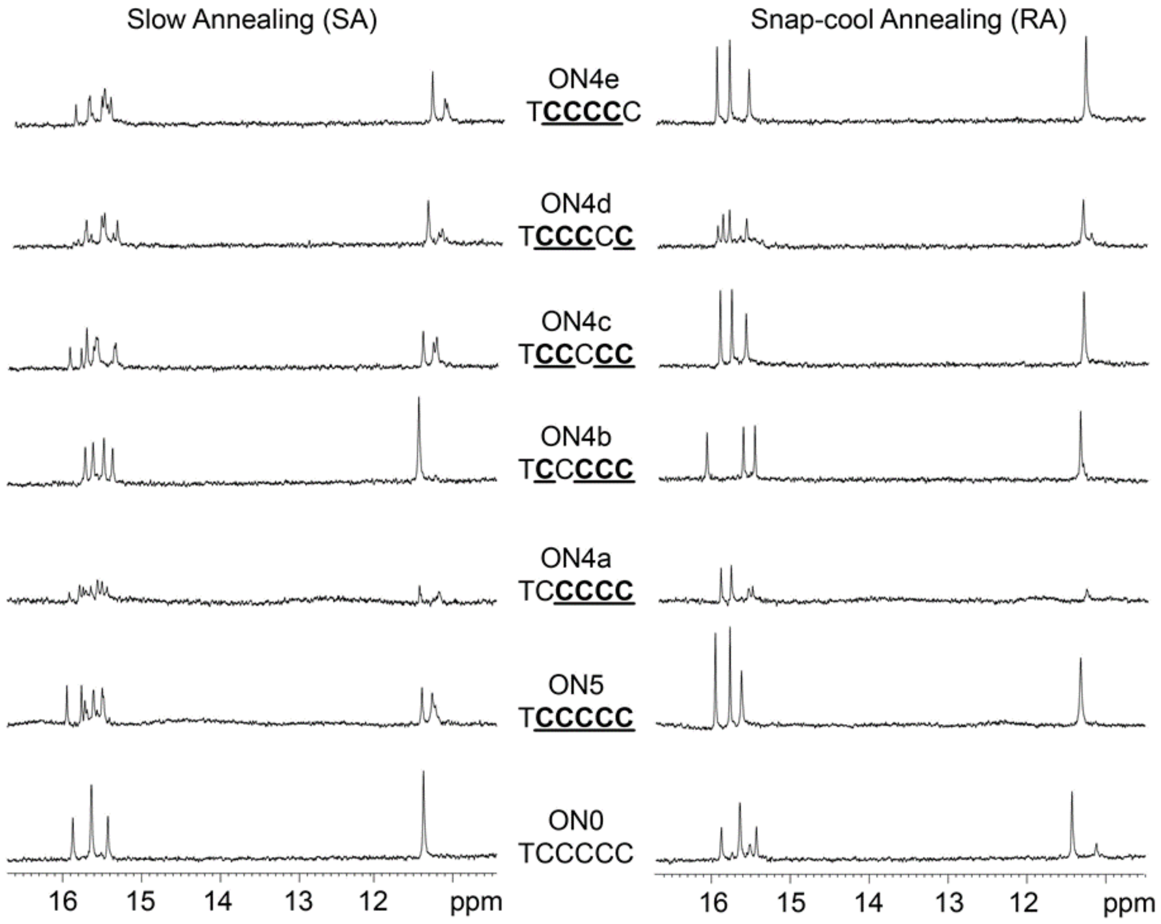

Supplementary Figure 6. Imino proton region of the ^{1}H NMR spectra of slow-annealed and rapid, fast-cooled (RA) ON0, ON5, and ON4a-ON4e (150 μM, 5°C).

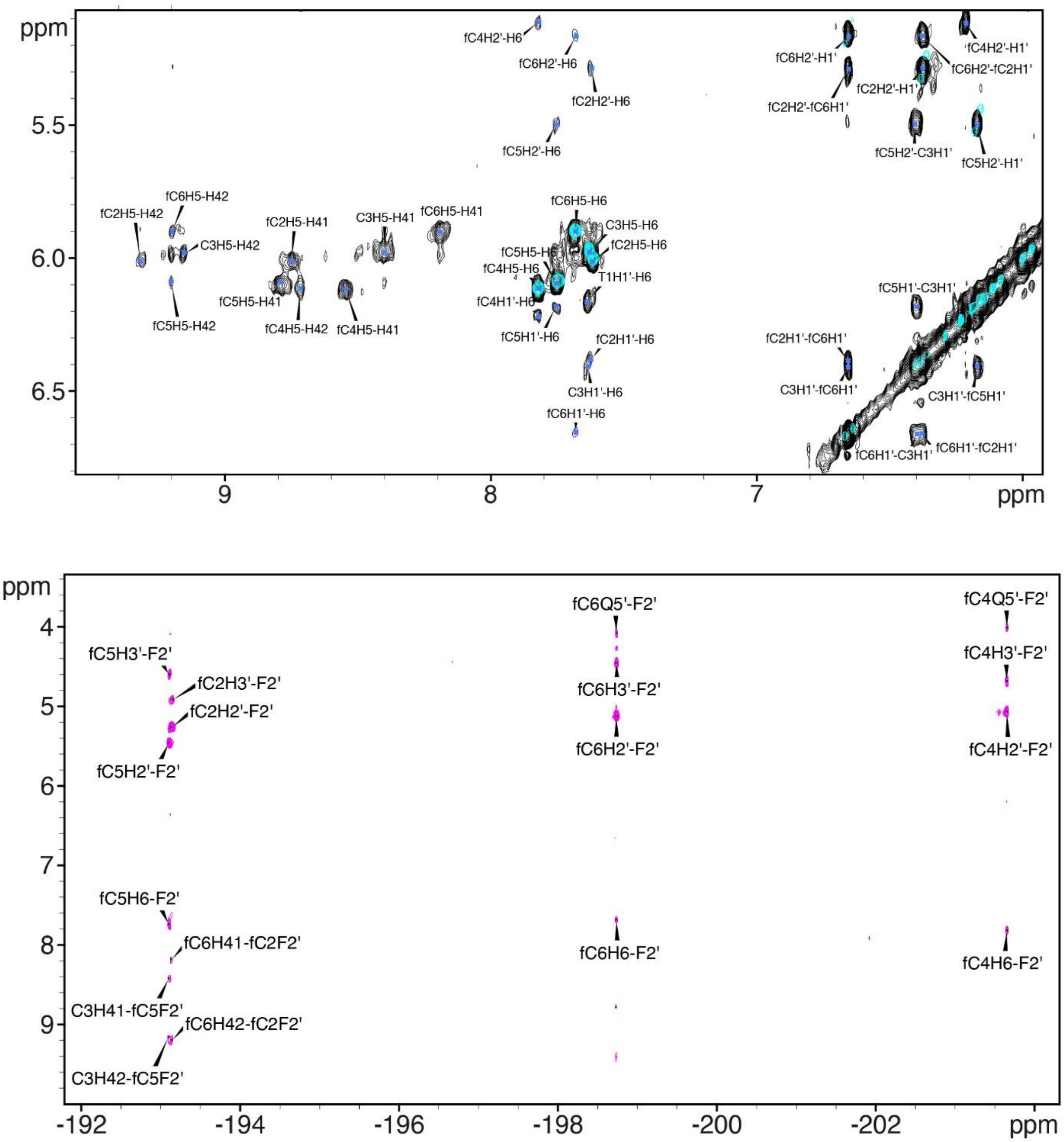

Supplementary Figure 7. Top. Selected region of the NOESY (black) and TOCSY (blue, overlapped) spectra of dimeric RA ON4b showing that it is folded into an i-motif structure. Bottom. ^{19}F-^{1}H HOESY spectrum of RA ON4b. Sample: 150 μM oligonucleotide in 10 mM NaPi pH 5, 5°C.Sample: 150 μM oligonucleotide in 10 mM NaPi pH 5, 5°C.

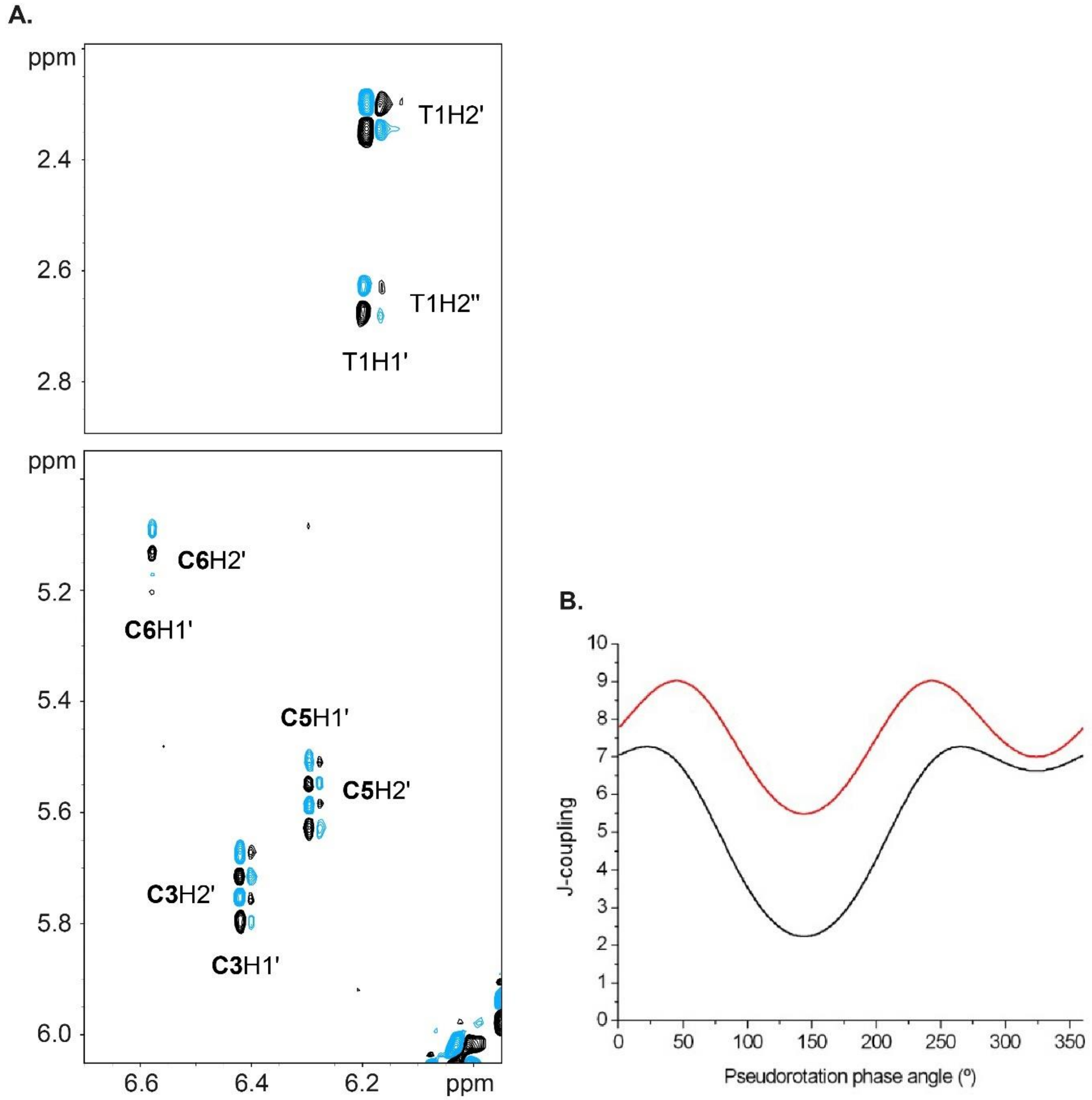

Supplementary Figure 8. (A) H1′-H2′ region of the DQF-COSY spectrum of rapid, snap-cooled (RA) ON5 (150 µM, 5°C). Cross-peaks for **C2** and **C4** are not observed, indicating that $J_{H1'-H2'}$ for these two residues are small. (B) Plot of $J_{H1'-H2'}$ vs pseudorotation phase angle (P) according to Karplus equation (parametrized from ref.1). The curve of $J_{H1'-H2'}$ for 2′F-arabino sugar is shown in dark. Low values indicate that the sugar conformation is in the South domain (P between 140º and 180º). The equivalent curve for deoxyribose ($J_{H1'-H2''}$) is shown in red for comparison. In the 2′F-arabino sugar, the electronegative substituent provokes a significant decrease of the ^{1}H-^{1}H J-coupling.

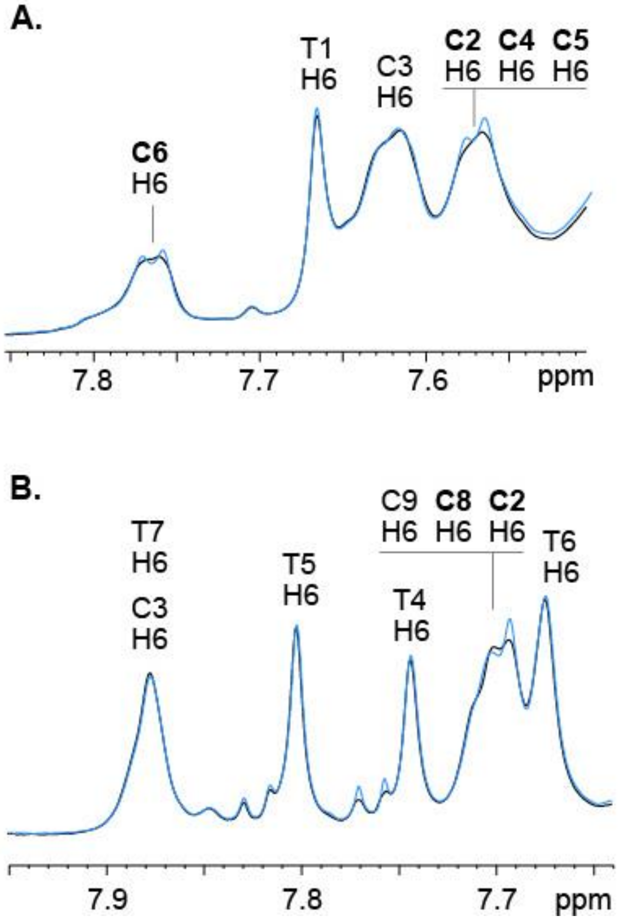

Supplementary Figure 9. Aromatic signals in the overlapped ^{19}F-coupled (black) and ^{19}F-decoupled (blue) 1D ^{1}H-NMR spectra of (A) tetrameric i-motif, SA ON4b (500 µM, 10 mM NaP_i pH 5, 5ºC) and (B) dimeric, centromeric i-motif, HC-3 (500 µM, 10 mM NaP_i pH 5, 5°C).

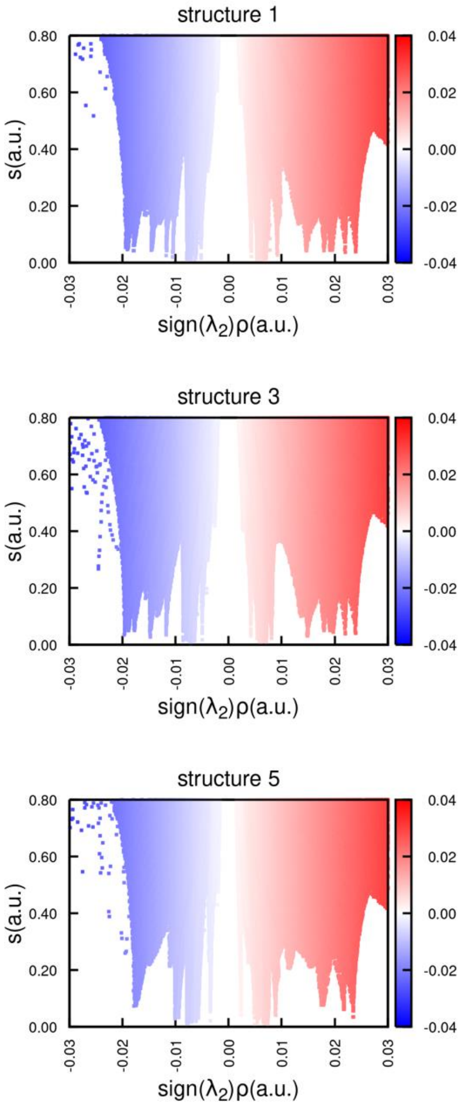

Supplementary Figure 10. NCI plots for residues **C6** and **C2′**.

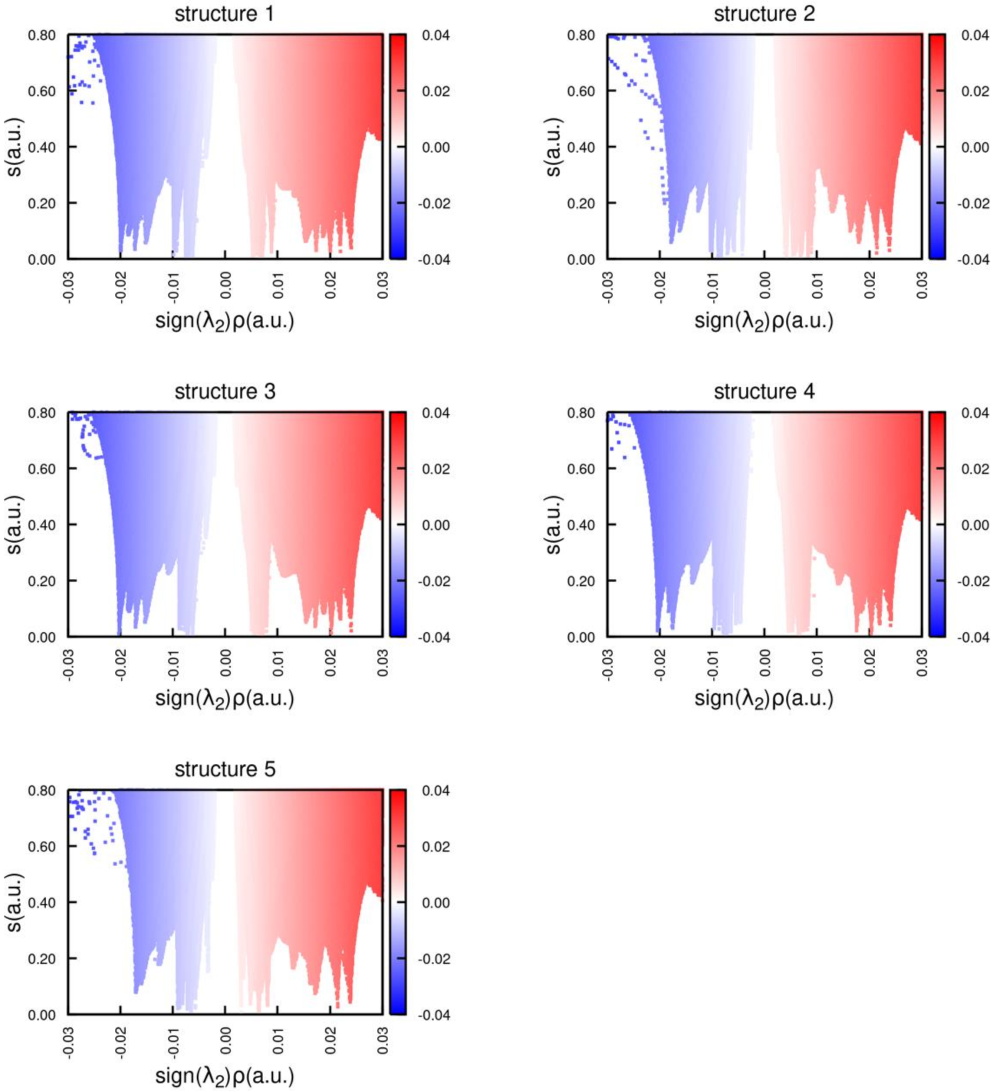

Supplementary Figure 11. NCI plots for residues **C2** and **C6′**.

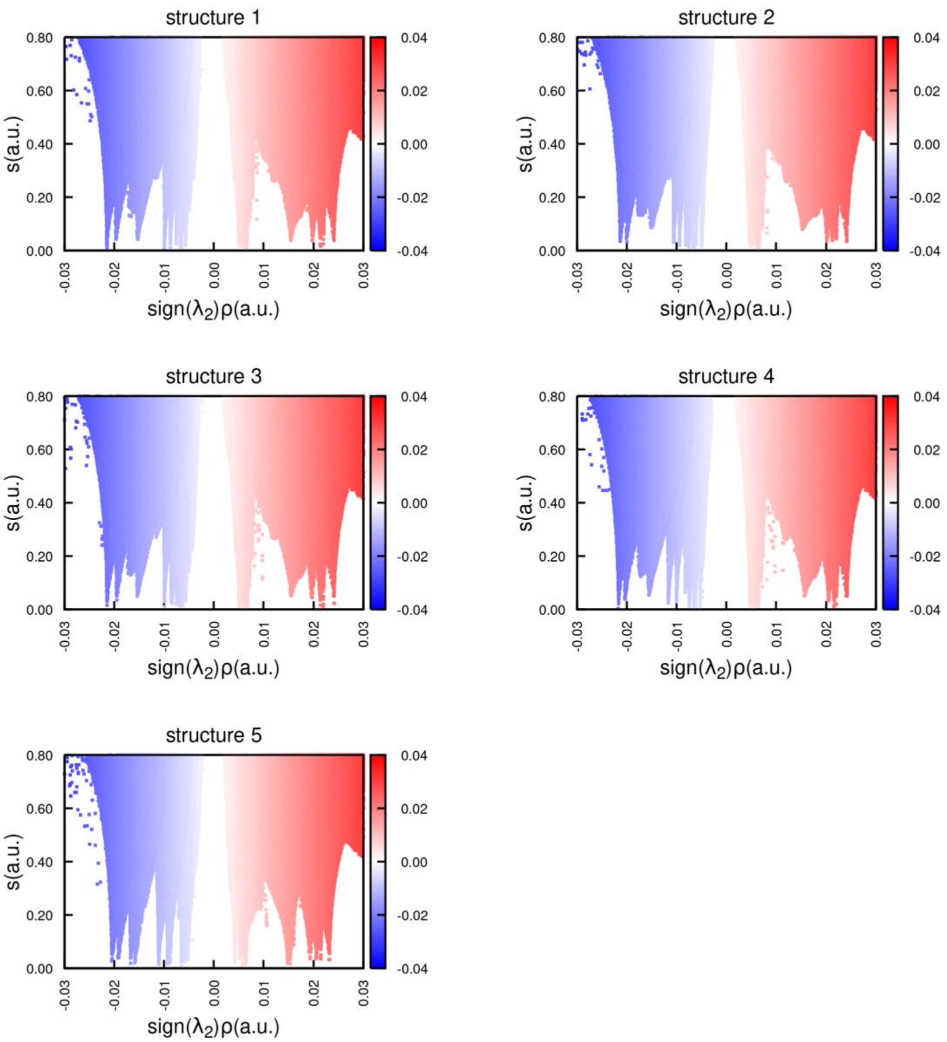

Supplementary Figure 12. NCI plots for residues **C3** and **C5′**.

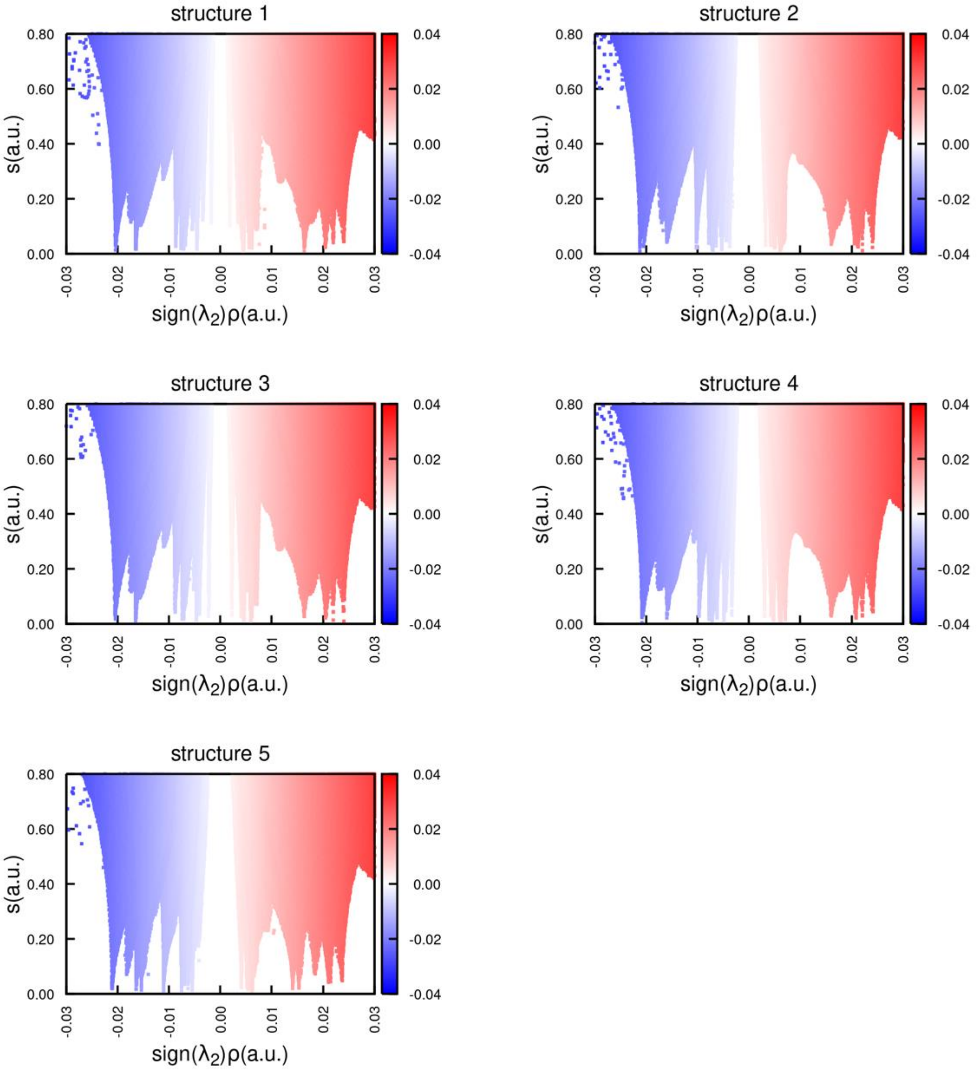

Supplementary Figure 13. NCI plots for residues **C5** and **C3′**.

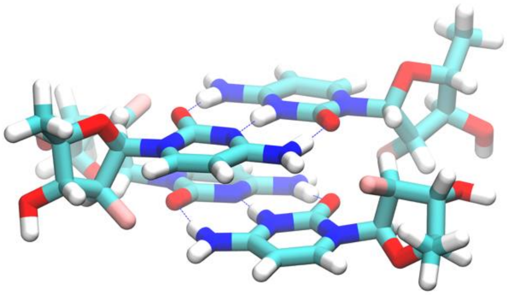

Supplementary Figure 14. Example of four-cytosine model system extracted from a MD dimeric i-motif structure and optimized. The backbone phosphate group was removed, the sugar 3′ ends are capped with a hydroxyl group, while the 5′ ends with a hydrogen atom.

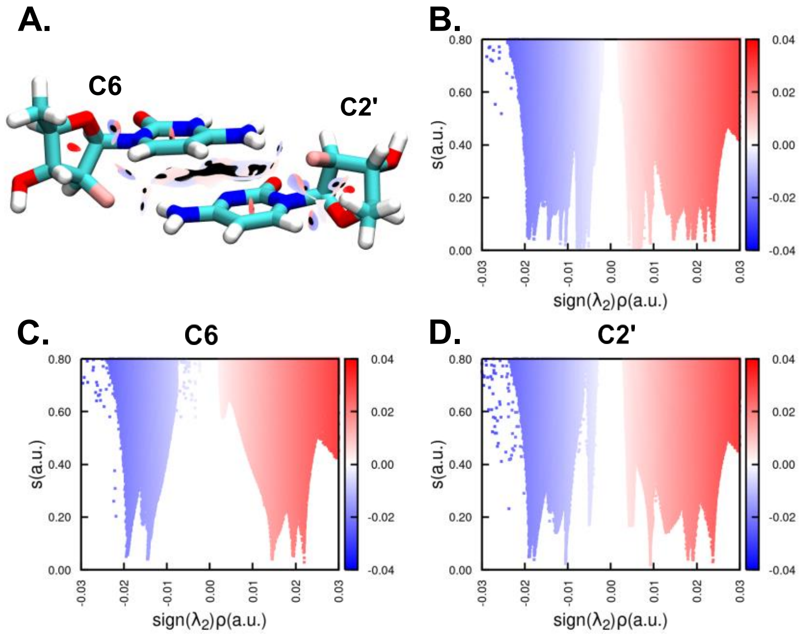

Supplementary Figure 15. NCI plots and reduced density gradient surface (s) for residues **C6** and **C2′**. (A) 0.04 au s isosurface colored as a function of ρ, and black 0.013 au s isosurface. Hydrogen bonds can be visualized as a blue s 0.04 isosurface (colored as in panels B-D) in correspondence of black spots (s =0.013). (B) NCI plots for **C6**-**C2′** two-residue system. (C,D) NCI plots for **C6** and **C2′** single-residue systems, respectively. Hydrogen bonds can be visualized as s blue spikes in the NCI plot regions (B-D) at sign(λ2)*ρ ≤ -0.01 au. NCI plots spikes in panel C-D are characteristic of intra-residual interactions, thus by comparing panels B and C-D we can discern intra- and inter-residual hydrogen bonds.

Assignment of spikes in NCI plots

Six spikes are recognizable in the **C6**-**C2′** NCI plot in the region sign(λ_2)*ρ ≤ -0.01 au (**Supplementary Figure 15B**). Five of them are intra-residual, as two and three of them appear in **C6** (**Supplementary Figure 15C**) and **C2′** (**Supplementary Figure 15D**) NCI plots, respectively. The two spikes in the **C6** plot can only be assigned to the isosurface between 2′F and H6, and between O2 and H1′, both displaying a s 0.013 isovalue spot. By observing the color of the 0.04 isosurface, we can assign the spikes at about -0.015 and -0.02 a.u. to 2′F···H6 and O2···H1′ interactions, respectively. Similarly, we can assign the O4′···H6 and the O2···H1′ of **C2′** residue to the spikes at about -0.02 and -0.01 a.u., respectively. The third spike at -0.018 au in the **C2′** plot is assigned to a spurious interaction involving the artificially introduced methyl group. Finally, the inter-residual **C6** 2′F···H4-2 **C2′** interaction is assigned to the spike at about -0.01 au. On the other hand, the **C2′** 2′F···H4-2 **C6** is not recognizable in the NCI plot, as located in a more crowded and weaker-interaction region (0 ≤ ρ ≤ -0.01 au).

Similar results may be found by analysis of **Supplementary Figures 17**-**19** for representative two-residue models **C2**-**C6′**, **C3**-**C5′**, and **C5**-**C3′.**

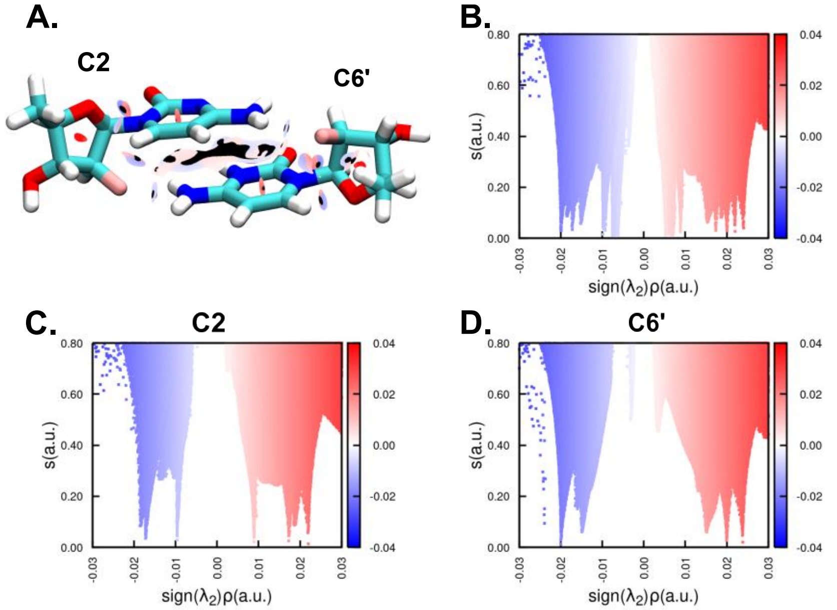

Supplementary Figure 16. NCI plots and reduced density gradient surface for residues **C2** and **C6′**. (A) 0.04 au s isosurface colored as a function of ρ, and black 0.013 au s isosurface. Hydrogen bonds can be visualized as a blue s 0.04 isosurface (colored as in panels B-D) in correspondence of black spots (s =0.013). (B) NCI plots for **C2**-**C6′** two-residue system. (C,D) NCI plots for **C2** and **C6′** single-residue systems, respectively. Hydrogen bonds can be visualized as s blue spikes in the NCI plot regions (B-D) at sign(λ2)*ρ ≤ -0.01 au. NCI plots spikes in panel C-D are characteristic of intra-residual interactions, thus by comparing panels B and C-D we can discern intra- and inter-residual hydrogen bonds. Details on the interpretation of the NCI plots are given in Section 7.1.

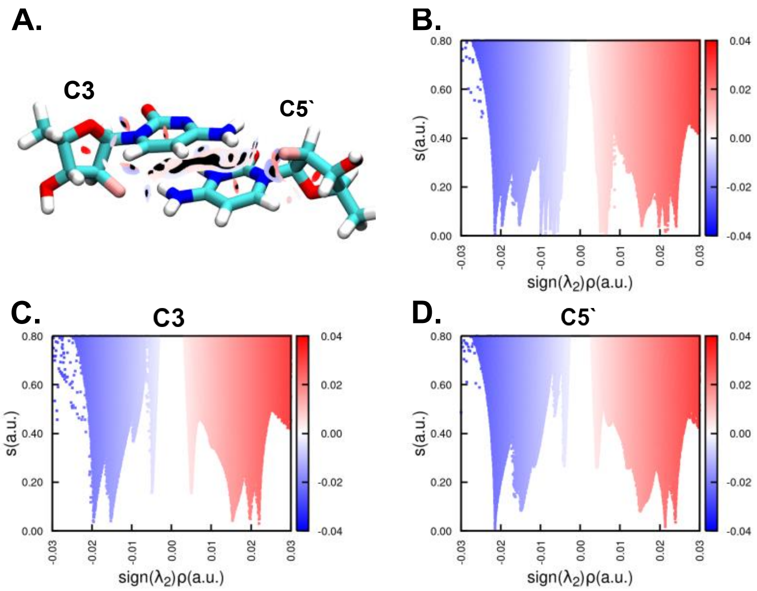

Supplementary Figure 17. NCI plots and reduced density gradient surface for residues **C3** and **C5′**. (A) 0.04 au s isosurface colored as a function of ρ, and black 0.013 au s isosurface. Hydrogen bonds can be visualized as a blue s 0.04 isosurface (colored as in panels B-D) in correspondence of black spots (s =0.013). (B) NCI plots for **C3**-**C5′** two-residue system. (C,D) NCI plots for **C3** and **C5′** single-residue systems, respectively. Hydrogen bonds can be visualized as s blue spikes in the NCI plot regions (B-D) at $\mathrm{sign}(\lambda 2)*\rho \leq -0.01$ au. NCI plots spikes in panel C-D are characteristic of intra-residual interactions, thus by comparing panels B and C-D we can discern intra- and inter-residual hydrogen bonds. Details on the interpretation of the NCI plots are given in Section 7.1.

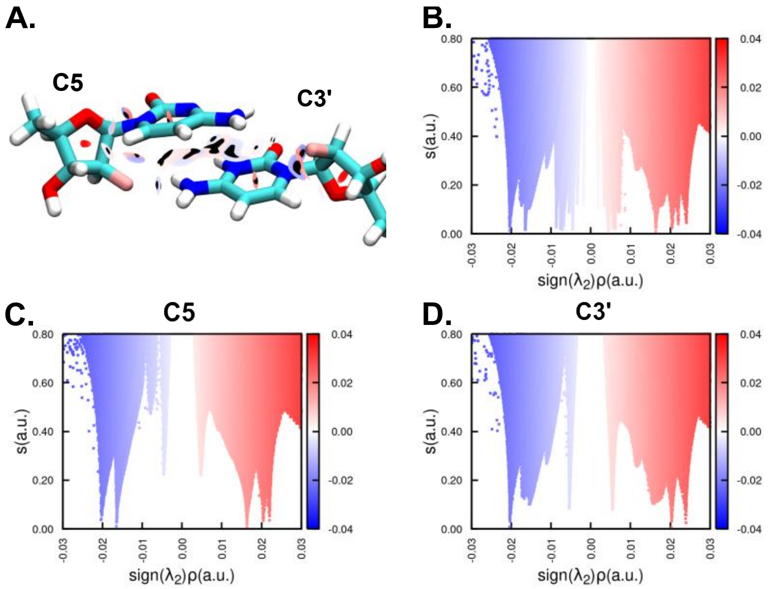

Supplementary Figure 18. NCI plots and reduced density gradient surface for residues **C5** and **C3′**. (A) 0.04 au s isosurface colored as a function of ρ, and black 0.013 au s isosurface. Hydrogen bonds can be visualized as a blue s 0.04 isosurface (colored as in panels B-D) in correspondence of black spots (s =0.013). (B) NCI plots for **C5**-**C3′** two-residue system. (C,D) NCI plots for **C5** and **C3′** single-residue systems, respectively. Hydrogen bonds can be visualized as s blue spikes in the NCI plot regions (B-D) at sign(λ2)*ρ ≤ -0.01 au. NCI plots spikes in panel C-D are characteristic of intra-residual interactions, thus by comparing panels B and C-D we can discern intra- and inter-residual hydrogen bonds. Details on the interpretation of the NCI plots are given in Section 7.1.

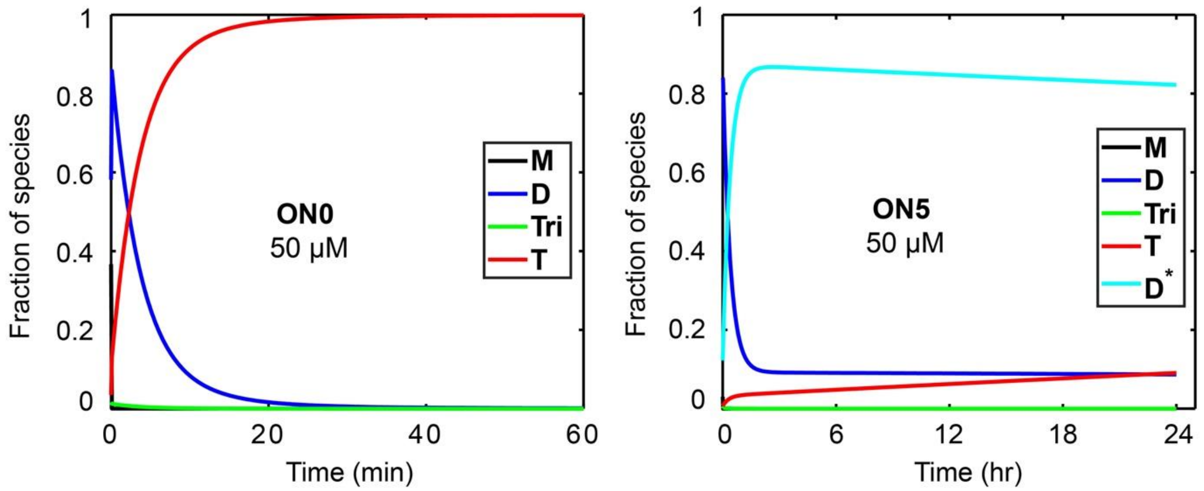

Supplementary Figure 19. Kinetic isothermal simulations of ON0 and ON5 at 4°C after cooling at 5°C/min.

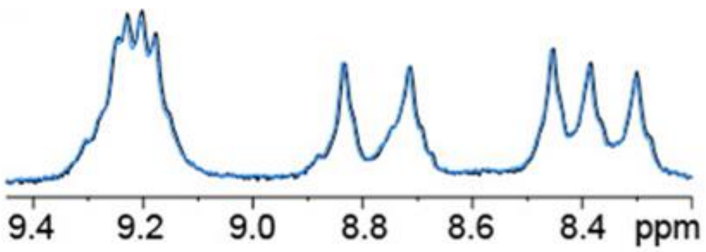

Supplementary Figure 20. Overlapped ^{19}F-coupled (black) and ^{19}F-decoupled (blue) 1D ^{1}H-NMR spectra showing amino proton signals of RA ON5.

B. Supplementary Tables

Supplementary Table 1. Chemical shifts of the proton and fluorine (F2′) signals in the respective ^{1}H and ^{19}F NMR spectra of rapid, fast-cooled (RA) ON5.

	H3	H42	H41	H6	H5/Me	H1′	H2′ [a]	H2′′	F2′	H3′	H4′	H5′/H5′′
T1	11.32	-	-	7.64	1.81	6.20	2.31	2.65	-	4.78	n.o.	n.o.
**C2**	n.o.	9.25	8.76	7.67	5.23	6.33	5.23	-	-194.5	n.o.	4.19	n.o.
**C3**	15.76	9.23	8.39	7.57	5.94	6.41	5.73	-	-189.3	4.92	4.17	n.o.
**C4**	n.o.	8.84	8.45	7.76	6.07	6.31	5.13	-	-201.9	4.76	4.47	4.08
**C5**	15.95	9.18	8.72	7.70	6.04	6.30	5.56	-	-192.3	4.62	4.52	4.10
**C6**	15.61	9.20	8.30	7.69	5.94	6.57	5.15	-	-199.1	4.50	4.28	n.o.

[a] H2′ of araF-C residues is the equivalent atom of H2″ in deoxyribose residues.
n.o. Not observed.

Supplementary Table 2. Experimental constraints and calculation statistics of ON5 dimeric i-motif

Experimental distance constraints		
Total number	50	
intra-residue	14	
sequential	6	
range > 1	30	
RMSD (Å)		
all well-defined* bases		0.23 ± 0.1 Å
all well-defined* heavy atoms		0.6 ± 0.2 Å
backbone		0.8 ± 0.3 Å
all heavy atoms		0.6 ± 0.2 Å
Residual violations	Average	Range
Sum of violation (Å)	0.05	0.00 - 0.17
Max. violation (Å)	0.04	0.00 - 0.10
NOE energy# (kcal/mol)	13	11.3 – 13.7
Total energy (kcal/mol)	-988	-1015 - -954
# K_{NOE} = 20 kcal/(mol Å^2)		

Supplementary Table 3. Average pseudorotation parameters of the dimeric structure of ON5.

Residue	Phase	Amplitude	Puckering
T1	146	43	C2'-endo
**C2**	162	38	C2'-endo
**C3**	35	39	C3'-endo
**C4**	180	40	C2'-endo
**C5**	33	38	C3'-endo
**C6**	41	37	C4'-exo
T1'	155	39	C2'-endo
**C2'**	143	35	C1'-exo
**C3'**	36	40	C3'-endo
**C4'**	181	40	C2'-endo
**C5'**	33	38	C3'-endo
**C6'**	49	36	C4'-exo

Supplementary Table 4. Average dihedral angles and order parameters (O.P.) of the dimeric structure of ON5.

Residue	α		β		Y		δ		ε		X		θ	
	Average	O.P.	Average	O.P.	Average	O.P.	Average	O.P.	Average	O.P.	Average	O.P.	Average	O.P.
T1	-	-	-	-	63	0,7	139	1,0	-154	1,0	-93	1,0	-78	1,0
**C2**	-94	1,0	58	1,0	178	1,0	148	1,0	-106	1,0	-141	1,0	-89	1,0
**C3**	90	1,0	-161	1,0	-170	1,0	81	1,0	-152	1,0	-111	1,0	-86	0,9
**C4**	-76	0,6	71	0,6	-164	1,0	154	1,0	-180	1,0	-129	1,0	-131	0,8
**C5**	-66	0,4	42	0,5	-172	1,0	80	1,0	177	1,0	-103	1,0	-79	1,0
**C6**	-58	1,0	175	1,0	65	1,0	79	1,0	-	-	-126	1,0	-	-
T1′	-	-	-	-	49	0,7	138	1,0	-168	1,0	-101	1,0	-87	1,0
**C2′**	-81	1,0	117	0,6	114	0,5	127	0,9	-66	0,4	-135	1,0	-92	0,4
**C3′**	13	0,4	-180	0,8	138	0,5	76	1,0	-154	1,0	-109	1,0	-79	1,0
**C4′**	-78	0,8	77	0,8	-167	1,0	154	1,0	179	1,0	-133	1,0	-155	0,8
**C5′**	-1	0,3	1	0,4	-172	1,0	78	1,0	179	1,0	-104	1,0	-79	1,0
**C6′**	-58	1,0	175	1,0	67	1,0	82	1,0	-	-	-129	1,0	-	-

Supplementary Table 5. Kinetic and thermodynamic parameters from globally fitting TH traces for i-motif assembly.

Parameter	ON0 Model 1	ON0 Model 2	ON5 Model 1	ON5 Model 1
Ea_1 (kJ/mol)	-0.5 ± 4	-0.5 ± 4	-63 ± 4	-64 ± 4
k_1 (M^{-1} min^{-1})	1.39e4 ± 0.08e4	1.22e4 ± 0.08e4	3.8e4 ± 0.3e4	4.9e4 ± 0.3e4
Ea_{-1} (kJ/mol)	123 ± 4	126 ± 3	140 ± 5	118 ± 4
k_{-1} (min^{-1})	5.7 ± 0.3	5.5 ± 0.3	1.4e-2 ± 0.2e-2	4.3e-2 ± 0.3e-2
Ea_2 (kJ/mol)	31 ± 2	32 ± 2	28 ± 2	28 ± 2
k_2 (M^{-1} min^{-1})	830 ± 40	920 ± 40	1.7e3 ± 0.1e3	300 ± 30
Ea_{-2} (kJ/mol)	97 ± 5	94 ± 4	106 ± 5	95 ± 5
k_{-2} (min^{-1})	2.3e-2 ± 0.2e-2	2.4e-2 ± 0.2e-2	28 ± 2	23 ± 2
Ea_3 (kJ/mol)	-42 ± 3	-40 ± 2	63 ± 5	69 ± 5
k_3 (M^{-1} min^{-1})	9.3e3 ± 0.6e3	9.7e3 ± 0.8e3	6.5e4 ± 0.6e4	1.8e5 ± 0.1e5
Ea_{-3} (kJ/mol)	355 ± 3	354 ± 3	278 ± 2	293 ± 2
k_{-3} (min^{-1})	9.7e-7 ± 0.8e-7	1.11e-6 ± 0.08e-6	1.7e-7 ± 0.1e-7	8.5e-8 ± 0.6e-8
Ea_F (kJ/mol)	-	-0.5 ± 12	-	-14 ± 1
k_F (min^{-1})	-	5.1e5 ± 0.8e-5	-	2.4e-2 ± 0.2e-2
Ea_U (kJ/mol)	-	100 ± 16	-	55 ± 3
k_U (min^{-1})	-	0.13 ± 0.02	-	2.1e-2 ± 0.2e-2
RSS	0.0091	0.0091	0.0063	0.004

C. Supplementary Methods

Analysis of TH Profiles:

The TH profiles were globally fit to two different assembly models as follows:

Sequential tetrameric assembly (Scheme A): Step-wise association of monomers (M), to dimers (D), to trimers (Tr) and finally to tetramers (T).

The changes in concentration with respect to time are:

$$\frac{d[M]}{dt} = 2 * k_{-1}[D] + k_{-2}[Tr] + k_{-3}[T] - [M] * (2 * k_1 * [M] + k_2 * [D] + k_3 * [Tr]) \quad \textbf{(1)}$$

$$\frac{d[D]}{dt} = k_1[M]^2 + k_{-2}[Tr] - [D] * (k_{-1} + k_2[M]) \quad \textbf{(2)}$$

$$\frac{d[Tr]}{dt} = k_2[D][M] + k_{-3}[T] - [Tr] * (k_{-2} + k_3[M]) \quad \textbf{(3)}$$

$$\frac{d[T]}{dt} = k_3[Tr][M] - k_{-3}[T] \quad \textbf{(4)}$$

Sequential tetrameric assembly with a folded dimer (Scheme B): Step-wise association of monomers (M), to dimers (D), to trimers (Tr) and finally to tetramers (T), with the possibility for the dimer (D) to collapse intramolecularly into a folded dimer (D^*).

The changes in concentration with respect to time are:

$$\frac{d[M]}{dt} = 2 * k_{-1}[D] + k_{-2}[Tr] + k_{-3}[T] - [M] * (2 * k_1 * [M] + k_2 * [D] + k_3 * [Tr]) \quad \textbf{(5)}$$

$$\frac{d[D]}{dt} = k_1[M]^2 + k_{-2}[Tr] - [D] * (k_{-1} + k_2[M]) - k_F[D] + k_U[D^*] \quad \textbf{(6)}$$

$$\frac{d[Tr]}{dt} = k_2[D][M] + k_{-3}[T] - [Tr] * (k_{-2} + k_3[M]) \quad \textbf{(7)}$$

$$\frac{d[T]}{dt} = k_3[Tr][M] - k_{-3}[T] \quad \textbf{(8)}$$

$$\frac{d[D^*]}{dt} = k_F[D] - k_U[D^*] \quad \textbf{(9)}$$

The rate constants are assumed to be functions of temperature following an Arrhenius relationship. The temperature dependences of the rate constants are given by:

$$k = k_0 e^{\frac{E_a}{R} * \left(\frac{1}{T_{ref}} - \frac{1}{T}\right)} \quad \textbf{(5)}$$

where k_0 is the rate constant at the reference temperature T_{ref} and E_a is the activation energy. In the global fit of the TH profiles, the set of equations **(1-4)** were numerically integrated using the ordinary differential equation (ODE) solvers in MATLAB to obtain the concentrations of monomer, dimer, trimer, and tetramer as a function of temperature. The absorbance profiles of the monomer and tetramer were assumed to be linear with temperature.

$$Abs_{cal(T,\mathrm{x})} = m_M * [M] + b_M + m_T * \left(\frac{[D]}{2} + \frac{3[Tr]}{4} + [T]\right) + b_T \quad \textbf{(6)}$$

The sets of TH profiles were fit by varying the kinetic parameters to minimize the RSS between the experimental and fitted absorbance data according to

$$RSS = \sum\left(Abs_{\exp(T)} - Abs_{calc(T,\mathrm{x})}\right)^2 \quad \textbf{(7)}$$

where $Abs_{exp(T)}$ and $Abs_{calc(T)}$ are the experimental and fitted absorbance profiles respectively, x = $[k_1, k_{-1}, k_2, k_{-2}, k_3, k_{-3}, k_F, k_U, E_1, E_{-1}, E_2, E_{-2}, E_3, E_{-3}]$ are the rate constants at the reference temperature and activation energies governing assembly and disassembly of the tetramer. Both 50 µM and 250 µM profiles at 0.5 and 5°C/min were fit together. Errors for fitted parameters were calculated using a bootstrapping approach,[2] in which each bootstrap sample was obtained by random resampling of the original data. For example, if the original dataset contained N points, each bootstrap sample was constructed by randomly selecting N of these data points, such that points may be selected more than once or not at all. 500 bootstrap samples were constructed and fitted using the kinetic model described above. The errors in the extracted parameters were taken as the standard deviations of the 500 sets of parameters obtained for all bootstrap samples.

D. Supplementary References

1. Wijmenga, S. S.; Mooren, M. M. W.; Hilbers, C. W., NMR of nucleic acids; from spectrum to structure. In *NMR of Macromolecules. A Practical Approach*, Roberts, G. C. K., Ed. IRL Press: New York, 1993; pp 217.

2. Hutcheon, J. A.; Chiolero, A.; Hanley, J. A., Random measurement error and regression dilution bias. *BMJ* **2010,** *340*, c2289.
